# Effects of a traditional Chinese medicine formula containing the *Coix seed* and *Lotus seed* on the intestinal morphology and microbiota of local piglets

**DOI:** 10.1186/s13568-021-01318-1

**Published:** 2021-11-27

**Authors:** Zhaolong Li, Zhongning Lin, Zheng Lu, Zhaoyang Ying

**Affiliations:** 1 Institute of Animal Husbandry and Veterinary Medicine, Fujian Academy of Agricultural Sciences, Pudang, Jin-an District, Fuzhou, 350013 Fujian Province China; 2 Agricultural Ecology Institute, Fujian Academy of Agricultural Sciences, Pudang, Jin-an District, Fuzhou, 350013 Fujian Province China

**Keywords:** Traditional Chinese Medicine Formula, Piglets, Gut microbiota, pH, Villus, Production performance

## Abstract

A traditional Chinese medicine formula based on the *Coix seed* and *Lotus seed* has been used as a general treatment of malnutrition, excessive fatigue, dysfunction of the spleen and stomach, and disorders of water transport in humans in China. However, there is limited information on its effects on the gut microbiota of piglets in vivo. In this study, the mix of *Coix seed* and Lotus were added the diet of forty weaned piglets (local piglets), and then evaluated it’s affected on the gut microbiota of piglets and on the relations within the gut bacterial community. The results indicated that this traditional Chinese medicine formula (LM) and the extract of the traditional Chinese medicine formula (LMT) downregulated pH of succus gastricus and raised pH of the ileum, and LMT obviously decreased the feed conversion ratio. Further study showed LMT and LM also significantly increased the thick and long of gastrointestinal villi. And then, 16S ribosomal DNA sequencing revealed that groups LMT and LM have higher relative abundance of the genus *Lactobacillus* in the colon, succus gastricus, and jejunum, which are beneficial bacteria sold as dietary supplements to aid digestion or to augment health. Meanwhile, the relative abundance levels of *Prevotellaceae*, *Alloprevotella*, and *Prevotella* in the colon and *Clostridium* in succus gastricus and jejunum were lower. These experiments highlight the usefulness of the traditional Chinese medicine formula based on the *Coix seed* and *Lotus seed* for decreasing pH in succus gastricus, for improving the structure of intestinal villi and gut microflora, and then for achieving improvements in pig production performance.

## Introduction

Traditional Chinese medicine (TCM) is an integrative result of various bioactive compounds and shows fewer adverse effects and excellent therapeutic actions and involves a broad range of medicinal practices (Normile et al., 2003; Wang et al., 2002) , which have been developed in China for thousands of years.

*Coix seed*s are mature kernels of *Coix lacryma-jobi L. var. ma-yuen* (Roman.) *Stapf* (Fan et al., 2017), and *Lotus seed*s are mature seeds of *Nelumbo nucifera Gaertn* (Yi et al., 2015), which are rich in starch, lipids, proteins, polysaccharides, polyphenols, phytosterols, lactams, and other bioactive ingredients and have numerous physiological and pharmacological effects fortifying the spleen; inhibiting dampness, oxidative stress, and inflammation; and regulating endocrine and immune functions (Hidaka et al., 1992; Yen et al., 2005a, b; Zhang et al., 2014; Wu et al., 2013). Accordingly, *Coix seed*s and *Lotus seed*s have pharmaceutical and food value and are widely used in China and other countries as a dietary supplement for daily health maintenance (Shao et al., 2009). Previously, research has demonstrated that some TCM agents can cause significant alterations of the human gut microflora and restore the balance of the gut ecosystem by inhibiting the growth of certain nonculturable bacteria and promoting proliferation of probiotics (Li et al., 2010; Zu et al., 2016). In recent years, some studies revealed that oligosaccharides and resistant starch of Lotus can stimulate the growth of *Lactobacillus* and *Bifidobacterium* as well as *Lachnospiraceae*, *Ruminococcaceae*, and *Clostridium*, respectively, and inhibit the growth of *Rikenellaceae* and *Porphyromonadaceae* in the human gut (Zhang et al., 2013, 2014; Zeng et al., 2018). Even though the *Coix seed* is similar to the *Lotus seed* and contains starch as its main component, there is limited information on the effects of the ingredients of the *Coix seed* on the in vivo gut microbiota (Li et al., 2019 and Liu et al., 2019), and thus, few studies have focused on the effects of a TCM formula based on the *Coix seed* and *Lotus seed* on gastrointestinal microbes of piglets.

The objective of this study was to reveal the effects of the TCM formula based on the *Coix seed* and *Lotus seed* on production performance, the structure of gastrointestinal villi, the gut microbiota of piglets, and the relations within the gut bacterial community.

## Materials and methods

### Animals and experimental design

Forty weaned piglets (local piglets), with weights of 7.33 ± 0.03 kg (certification number: 2020000335580), were bought from Nangping Co., Ltd. and were randomly subdivided into four groups (LC, LK, LM, and LMT). The control group (LC, n = 10), was fed the basal diet. The piglets in the antibiotic group (LK) were provided fodder containing 1/10000 amoxicillin powder for animals (LK group, n = 10). The piglets in the TCM formula (LM) powder group consumed fodder containing 5/100 TCM formula powder (Table 1; LM group, n = 10). The piglets in the TCM formula extract group were fed with fodder containing 1/100 of the TCM formula extract (LMT group, n = 10). The extraction process as follows: (1) Extract: The TCM formula was added to the extraction tank, soaked in drinking water at 6 times the weight of the drug for 12 h, and cooked twice (boiling, temperature 95–100℃), water was added at 6 times of the drug weight and volume), 1.5 h at each time, the extract was filtered twice. (2) Concentration: The extract was concentrated at a relative density of 1.10 at 50℃ by mixing the filtrated extract collected in step 1; (3) Drying: dextrin (1% by weight) was added to the concentrated liquid, which was stirred evenly and dried by spraying; the temperature in the drying tower was 90–95 ℃. (4) Sieve mixing: The spray-dried powder was passed through an 80–160 mesh sieve, mixed evenly, and packaged to be used in the next step. The piglets were housed in a room with a controlled temperature (23 °C ± 1 °C), relative humidity (60 − 65%), and 12 h light/ dark cycle (lights on at 8:00 a.m. and off at 8:00 p.m.). and all diets started on day 1 and ended on day 28. All the piglet experiments conducted in this study were in compliance with the Guidelines for the Care and Use of Laboratory Animals published by the U.S. National Institutes of Health (NIH Publication 85-23, 1996), and all the procedures were approved by the Animal Care Review Committee (Approval No. 2020000335580), Fujian Academy of Agricultural Sciences, China. The basal diet was purchased from Fujian Pajie Organism Co., Ltd. (Fujian, China). The compositions of the five diets are presented in Table 1, and nutrition components were in accordance with GB 14924.3-2010 standards (China).

**Table 1:** Basic diet and nutrients

	5–30 kg
Corn /%	50
Macpi /%	4
Soybean meal /%	30
Fish Powder /%	6
Premix /%	10
Total /%	100
Digestive Energy (DE/) (MJ·Kg^−1^)	13.87
Crude-protein (CP/%)	19.5
Lysine acid (Lys/%)	1.1
Calcium (Ca /%)	0.9
Effective phosphorus (AP/%)	0.45

### Determination of the average body weight and FCR

All piglets were weighed after overnight fasting on the morning of day 1 and on days 14 and 28 of the feeding experiment. The feed intake of each piglets was recorded daily, and then throughout the experiment to calculate the FCR.

### Measurement of pH in the gastrointestinal tract

At the end of the 28 d experiment, the piglets were euthanized in accordance with the experimental animal procedures approved by the Institutional Ethics Committee/Animal Care and Use Committee, and pH of succus gastricus, of the duodenum, small intestine, colon, ileum, cecum, and rectum contents were measured with a pH meter (PHS-5C PH, Guangzhou, China), and then statistical analysis of the recorded data was carried out.

### Gastrointestinal villi

Tissue samples (1–2 cm) of the stomach, duodenum, small intestine, colon, ileum, cecum, and rectum were collected from the piglets and fixed for 12 h in 5 mL of 4% paraformaldehyde. The fixed tissue was sequentially passed through 50%, 70%, 80%, 90%, 100%, and 100% ethanol, for 35 to 45 min at each concentration and was clarified in 100% ethanol + xylene (1:1) for 30 to 40 min. The tissue samples were then immersed in paraffin (1:1) for 30 min. Sections of 6–12 µm thickness were prepared (average 7–8 µm), placed on slides, and stained with hematoxylin and eosin. The sections were examined, and villus length was measured under a microscope.

### Sample collection and extraction of genomic DNA from gastrointestinal microorganisms

The contents of the stomach, duodenum, small intestine, colon, ileum, cecum, and rectum were collected from the piglets, and total genomic DNA from these samples was extracted by the cetyltrimethylammonium bromide (CTAB) and sodium dodecyl sulfate (SDS) method, and then DNA concentration and purity were examined on 1% agarose gels. According to the concentration, DNA was diluted to 1 ng/µL with sterile water.

### Extraction of DNA and PCR amplification

The samples of each above-mentioned organ of the gastrointestinal tract, approximately 100 mg, were pulverized in liquid nitrogen, resuspended in 30 mL of 0.1 mol/L phosphate-buffered saline (pH 7.0), and stirred well. The supernatant was obtained by centrifuged three times at 12,000×*g* for 5 min. Then, the supernatant was centrifuged at 12,000× *g* for 5 min at room temperature, and the bacterial pellets were collected. The pellets were resuspended in 3 mL of TE buffer (pH 8.0), and then the DNA was extracted with the Power Fecal DNA Isolation Kit (Mo Bio Laboratories, Inc.) according to the manufacturer’s instructions. The DNA samples extracted from piglets’ feces were amplified with the primer Pro 341 F (5’-CCTACGGGNBGCASCAG-3’) and Pro 805R (5’-GACTACNVGG GTATCTAATCC-3’) (Nadkarni et al., 2002). All PCRs were carried out in 30 µL reaction mixtures containing 15 µL of the Phusion® High-Fidelity PCR Master Mix (New England Biolabs), 0.2 µM of forward and reverse primers, and ~ 10 ng of template DNA. Thermal cycling was started with the initial denaturation at 98 °C for 1 min; followed by 30 cycles of denaturation at 98 °C for 10 s, annealing at 50 °C for 30 s, and elongation at 72 °C for 60 s; with final extension at 72 °C for 5 min. The raw sequencing data were uploaded into the NCBI Sequence Read Archive database (SRR16895036-47).

### Bioinformatics analysis

Paired-end reads were merged using FLASH (Magoc and Salzberg., 2011), which was designed to merge paired-end reads when at least some of the reads overlap (a read was generated from the opposite end of the same DNA fragment), and the splicing sequences were designated as raw tags. Quality filtering of the raw tags was performed under specific filtering conditions to obtain high-quality clean tags according to the QIIME quality control process (Bokulich et al., 2013). The tags were compared with the reference database (Gold database) via the UCHIME algorithm to detect chimeric sequences, which were later removed (Edgar et al., 2011; Haas et al., 2011). Operational taxonomic units (OTUs) were clustered at a 97% similarity cutoff in the UPARSE software (Edgar, 2013), and chimeric sequences were identified and removed using UCHIME. The taxonomic identity of each 16S rRNA gene sequence was analyzed by means of RDP Classifier against the SILVA (SSU115) 16S rRNA database at a confidence threshold of 70% (DeSantis et al., 2006). Sequences with higher than 97% similarity were assigned to the same OTU. A representative sequence for each OTU was screened for further annotation. OTU abundance data were normalized using a standard of the sequence number corresponding to the sample with the fewest sequences. Subsequent analysis of α-diversity was performed based on these output-normalized data. α-Diversity was analyzed as six indices: observed-species (OS), Chao1, Shannon, Simpson, ACE, and good’s coverage. β-Diversity was evaluated by principal component analysis (PCA), which was conducted at phylum and OTU levels, and the hierarchical clustering tree was constructed based on Unweight Unifrac distances. All these indices for our samples were calculated in QIIME (Version 2.0) and were displayed with the R software (Version 3.2.5). The differences in a dominant bacterial community among the groups were detected via Line Discriminant Analysis (LDA) effect size (LefSe). The biomarkers used in the present study had an effect size threshold of 3.0.

### Statistical analysis

To evaluate significance of the differences in the relative abundance levels of individual taxa between two groups, Metastats software (Version: 1.0) was utilized. LEfSe was used for the quantitative analysis of biomarkers within different groups. This method was designed to analyze data in which the number of species is much higher than the number of samples and to provide biological class explanations to determine statistical significance, biological consistency, and effect size estimates of the predicted biomarkers. To identify the differences in microbial communities between two groups, analysis of similarities and a multi-response permutation procedure were performed based on Curtis dissimilarity distance matrices.

## Results

### Effects of the TCM formula on piglets’ weight and feed efficiency

**Fig. 1:**
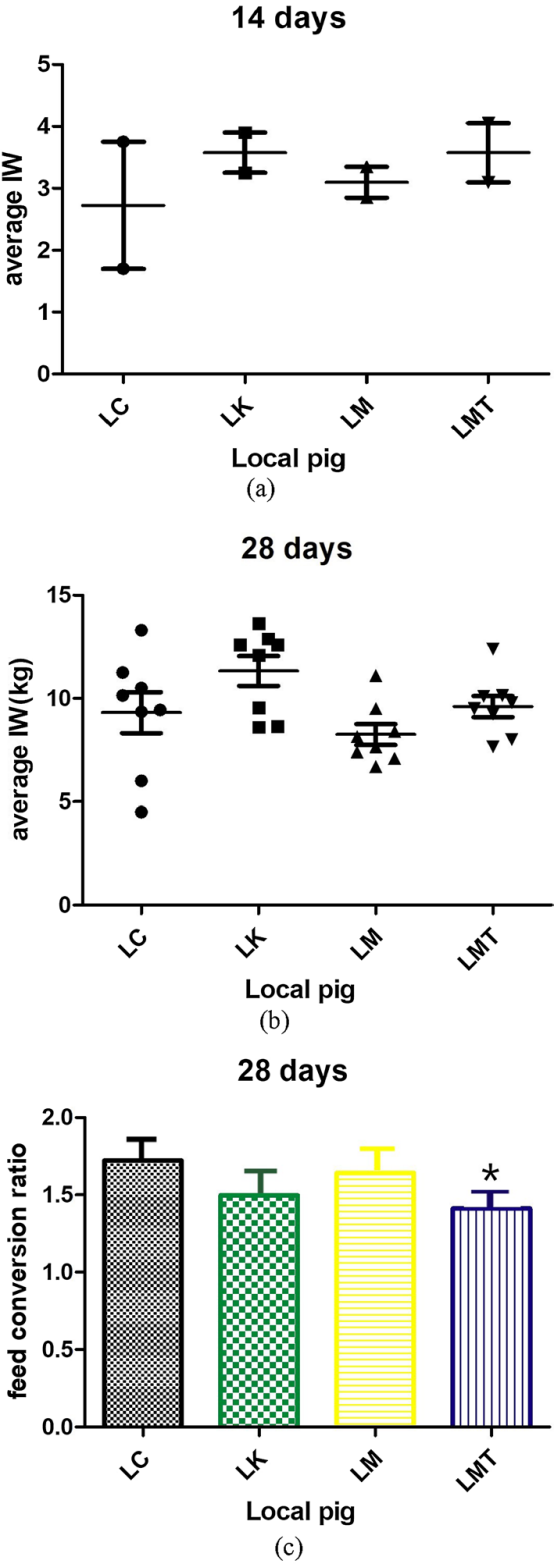
Average weight gain and FCR. **a** Average weight gain of weaned piglets at 14 d after the start of the feeding experiment. **b** Average weight gain at 28 d after the start of the feeding experiment. **c** FCR at 28 d of the feeding experiment (*p < *0.05*)

Throughout the experiments, piglets of the three treatment groups—antibiotic group (LK), TCM formula group (LM), and TCM formula extract group (LMT)—appeared healthier and livelier than those in the control (LC, no treatment) group. The fecal pellets were normal, and there were no deaths or diarrhea. Groups LK, LM, and LMT showed a higher average daily gain (ADG) during the 14d experimental period (Table 2). Following the next 14 d experimental period, the highest body weight was observed in the LK group, group LM showed the lowest weight, but no differences in the ADG of piglets were observed between groups LMT and LC (Fig. 1). The feed conversion ratio (FCR) was used to describe the relation between the total weight increment and the total feed intake of piglets. After the experimental period, the FCRs of piglets in groups LC, LK, LM, and LMT were 1.722 ± 0.013, 1.498 ± 0.018, 1.643 ± 0.021, and 1.412 ± 0.028, respectively (Table 2). The total feed intake in the LMT group was significantly lower than in groups LC, LK, and LM (*p* < *0.05*).

**Table 2:** Comparison of the growth performance of the local piglets

	LMT	LM	LK	Control
Total food intake (kg)	118.65	118.6	142.85	139.9
Average daily food intake (ADFI) (kg/per head)	0.42	0.42	0.51	0.50
Total additive addition amount (kg)	5.93	5.93	7.14	0
Actual additive ratio (%)	5	5	0.05	0
Initial weight (kg)	7.36 ± 0.58	7.36 ± 0.55	7.37 ± 0.78	7.38 ± 0.28
Last weight (kg)	15.76 ± 3.00	14.58 ± 2.34	16.9 ± 2.05	15.5 ± 2.54
Average weight gain (kg)	8.4 ± 1.24	7.22 ± 1.29	9.54 ± 2.84	8.13 ± 1.48
Average daily gain (ADG) (kg/per head)	0.30 ± 0.15	0.26 ± 0.34	0.34 ± 0.17	0.29 ± 0.10
Feed/Weight gain ratio (F/G)	1.412	1.643	1.498	1.722
F/G vs. control group comparison	↓0.31 (18%)	↓0.079 (4.6%)	↓0.224 (13.00%)

### Effects of the TCM formula on the gastrointestinal pH of the piglets

**Fig. 2:**
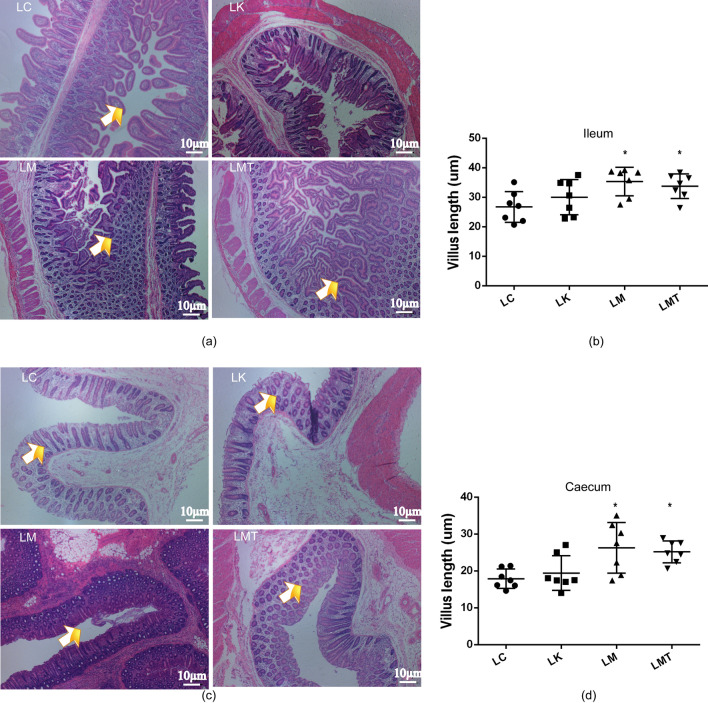
Variation in pH of the gastrointestinal content of weaned piglets on day 28. **a** pH levels of succus gastricus, **b** pH of rectal content, **c** pH of cecal content, **d** pH of ileal content, **e** pH of duodenal content, **f** pH of jejunal content, and **g** pH of colonic content (*p < *0.05*)

pH varies among different regions of the gastrointestinal tract in piglets and plays different roles in the physiological and biochemical processes. At the end of the 28d experimental period, compared with the LC (PH = 4.86) and LK group (PH = 4.91), groups LM (PH = 3.12, *p* < *0.05*) and LMT (PH = 3.35, *p* < *0.05*) had significantly lower pH in succus gastricus (Table 3), and higher pH in the ileum (LM: PH = 6.57, *p* < *0.01*; LMT*:* PH = 6.46, *p* < *0.01*), and the PH of jejunum among the LMT group also has higher than other groups (PH = 6.35, *p* < *0.05*). However, the pH value showed no significant differences in the caecum, colon, duodenum, and jejunum of the piglets among the three groups (Fig. 2).

**Table 3:** pH of the gastrointestinal content of weaned piglets on day 28

Site	LMT	LM	LK	LC
Caecum	6.26 ± 2.23	5.59 ± 0.98	5.64 ± 0.23	5.48 ± 0.23
Colon	5.86 ± 2.03	5.78 ± 2.79	5.9 ± 0.29	5.51 ± 1.36
Duodenal	6.14 ± 1.64	5.82 ± 2.22	5.74 ± 1.51	5.63 ± 1.56
Ileum	6.50 ± 1.15	6.66 ± 1.38	5.84 ± 1.16	5.59 ± 1.15
Succus gastricus	3.15 ± 1.84	3.36 ± 1.56	5.04 ± 1.49	5.01 ± 1.50
Jejunum	6.38 ± 1.89	5.87 ± 1.76	5.75 ± 1.62	5.47 ± 1.35

### Evaluation of the morphological features of gastrointestinal villi

**Fig. 3:**
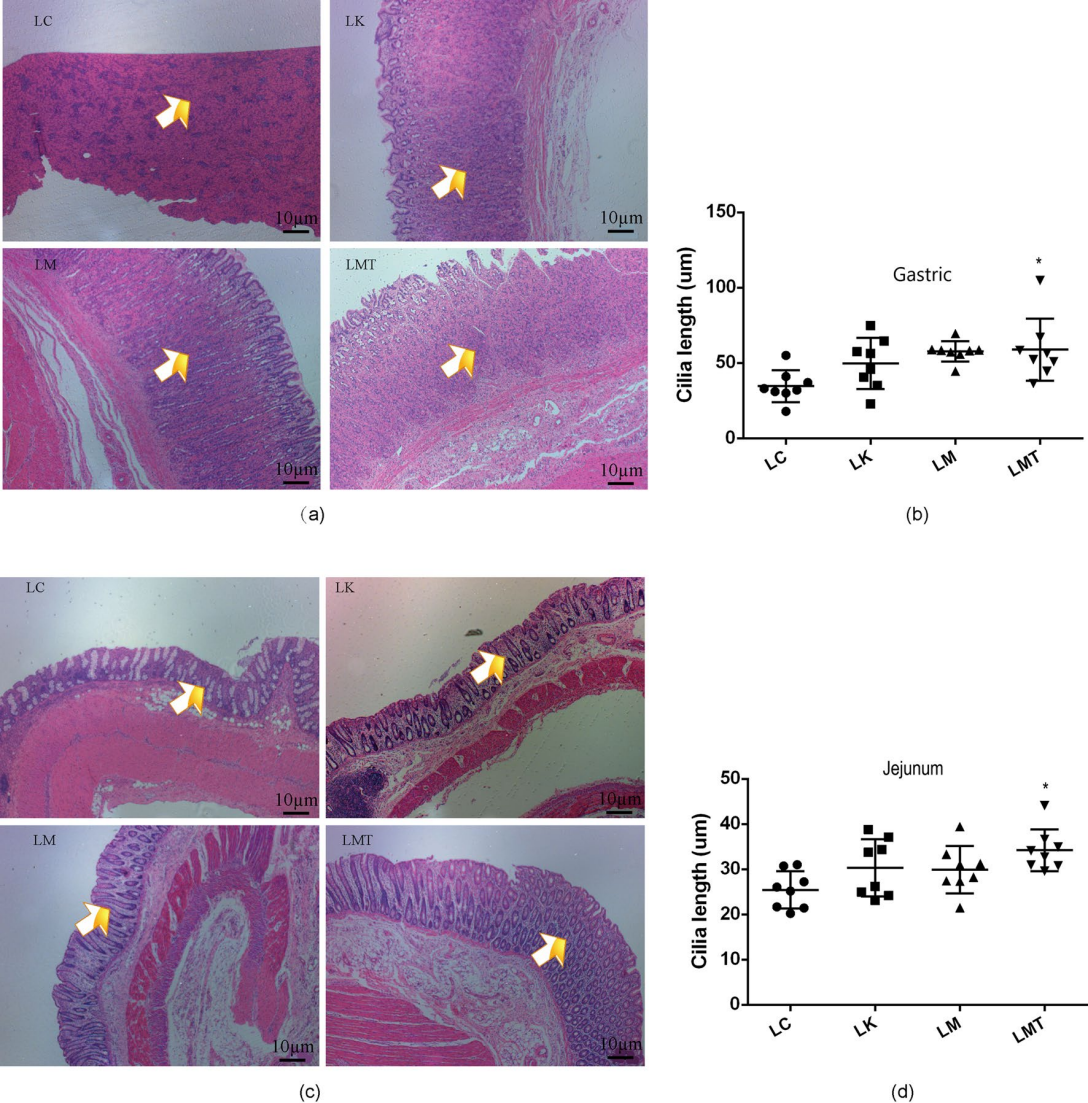
Morphological changes in gastrointestinal villi of the weaned piglets. **a** Morphological changes of gastric villi in the weaned piglets. **b** The length of gastric villi. **c** Morphological changes in the jejunum of the weaned piglets. **d** The lengths of jejunal villi in different groups (*p < 0.05; **p < 0.02; ***p < *0.01*)

**Fig. 4:**
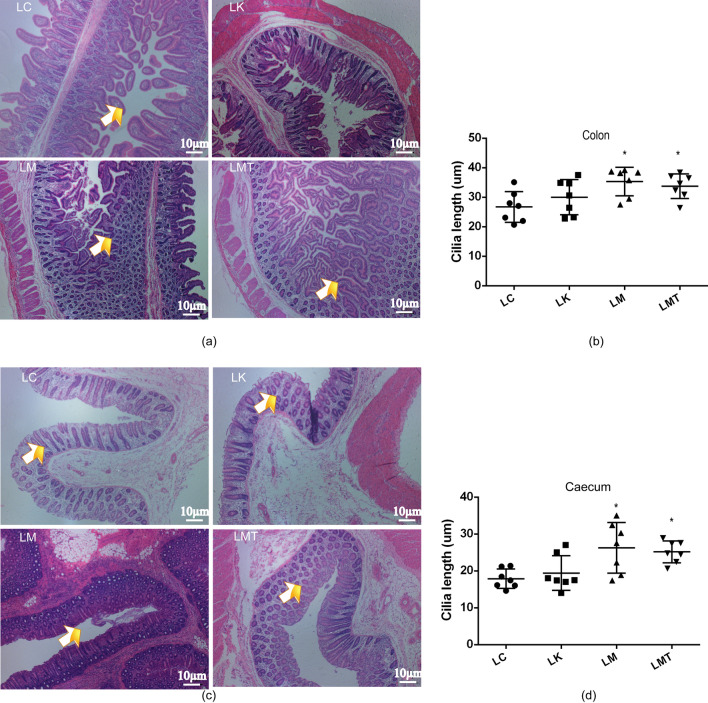
Morphological changes in the cecal and colonic villi of weaned piglets. **a** Morphological changes in the cecum of the weaned piglets. **b** Length of colon villi. **c** Morphological changes in the cecal of weaned piglets. **d** Length of colonic villi in different groups (* p < 0.05; ***p < *0.01*)

Gastrointestinal villi and microvilli not only increase intestinal absorptive surface area but also provide a micro-environment for the growth of the gut microbiota and promote the exceptionally efficient absorption of nutrients in the lumen. In this respect, groups LM and LMT showed greater length, width, and density of villi on the gastric wall and colon wall than did groups LC and LK (Fig. 3). In addition, the villi of the cecum wall and jejunum wall in groups LM and LMT also yielded similar results. These data suggest that the *Coix seed* and *Lotus seed* may stimulate intestinal villi growth in piglets (Fig. 4).

### Composition differences of gut microbiota among four groups

**Fig. 5:**
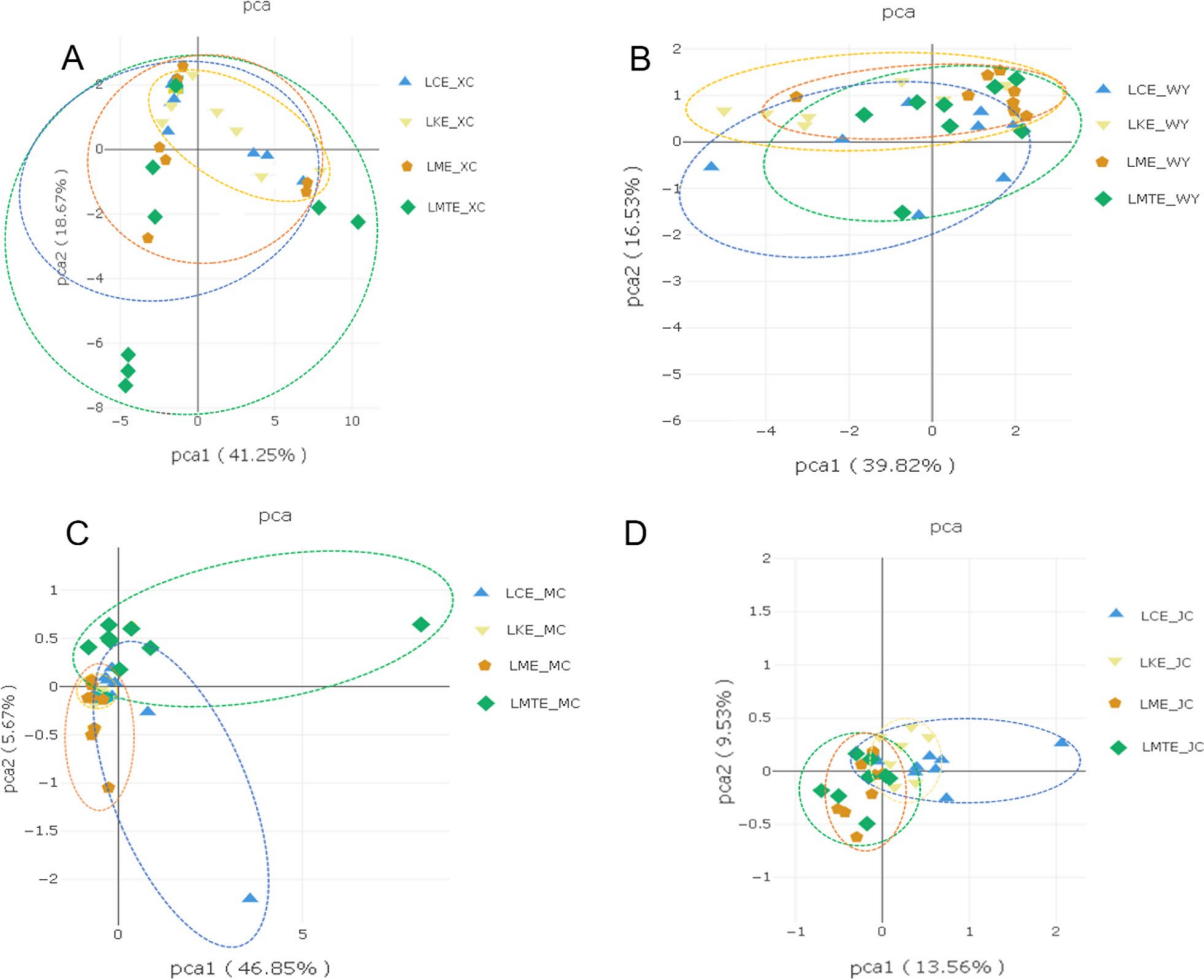
Bacterial community composition were different among LC, LK, LM and LMT. Principal coordinates analysis plots show unweighted UniFrac distances in four group piglets (PERMANOVA: unweighted R2 = 0.101, p = 0.001). Dots and surrounding dashed ellipses (95% confifidence level) represent the gut bacterial communities of LC (blue), LM (yellow), LK (brown) and LMT (green) piglets. **A** jejunum. **B** succus gastricus. **C** cecum. **D** colon

Sequencing analysis indicated that species richness of gut microbiota on LK group was lowest than other groups (Chao1 on colon: LK, 25,104.533 ± 109.407, LC, 28,094.745 ± 208.352; LM, 27,288.283 ± 21 3.735; and LMET, 26,326.204 ± 276.666) and other indexes showed similar results, such as Shannon (Colon of LK, 12.411 ± 1.375; Caecum of LK, 12.890 ± 1.627; Succus gastricus of LK, 10.967 ± 1.097; Jejunum of LK 9.752 ± 1.075), but the gastro-intestinal species richness of LC、LM and LMT groups shown higher (Table 4). Ace indexes also similar results. We used unweighted UniFrac distances to separate the LM、LMT and LK group. The PCoA explained 9.53 and 61.24% variation among four groups piglets for unweighted UniFrac distances, respectively (Fig. 5). The inter-individual beta diversity varied significantly (*p* < *0.001*) in all the four group piglets. Among TCM groups (LM and LMT group) shown higher beta diversity index than LK group, which indicates high level of variety in the microbial composition of LM and LMT group piglets (Fig. 5).

**Table 4:** Alpha diversity indices

Group	Shannon	Chao 1	Ace
*LCE_JC*	13.022 ± 1.253	28,094.745 ± 208.352	29,113.150 ± 256.423
*LKE_JC*	12.411 ± 1.375	25,104.533 ± 109.407	26,086.854 ± 258.355
*LME_JC*	12.655 ± 2.112	27,288.283 ± 213.735	28,431.517 ± 357.526
*LMTE_JC*	12.636 ± 1.143	26,326.204 ± 276.666	26,392.808 ± 278.177
*LCE_MC*	12.569 ± 1.009	26,411.583 ± 354.109	27,580.895 ± 286.396
*LKE_MC*	12.890 ± 1.627	27,295.407 ± 321.423	28,054.284 ± 439.329
*LME_MC*	12.834 ± 0.988	27,811.435 ± 432.767	28,716.256 ± 397.491
*LMTE_MC*	12.241 ± 1.172	24,683.871 ± 307.611	25,777.705 ± 303.776
*LCE_WY*	11.099 ± 1.053	16,422.965 ± 199.865	17,168.775 ± 245.980
*LKE_WY*	10.967 ± 1.097	17,937.138 ± 303.123	18,781.656 ± 277.313
*LME_WY*	11.911 ± 1.564	20,529.737 ± 206.639	21,043.125 ± 331.694
*LMTE_WY*	11.674 ± 2.053	20,441.081 ± 193.708	21,566.082 ± 425.101
*LCE_XC*	10.784 ± 1.122	13,793.828 ± 177.067	14,423.189 ± 222.039
*LKE_XC*	9.752 ± 1.075	9718.633 ± 252.278	9951.928 ± 355.228
*LME_XC*	10.176 ± 1.636	11,670.409 ± 351.185	12,139.562 ± 211.371
*LMTE_XC*	10.387 ± 1.405	13,518.902 ± 407.534	14,699.148 ± 197.012

### Comparison of the predominant bacterial community structure in the colon among the groups

**Fig. 6:**
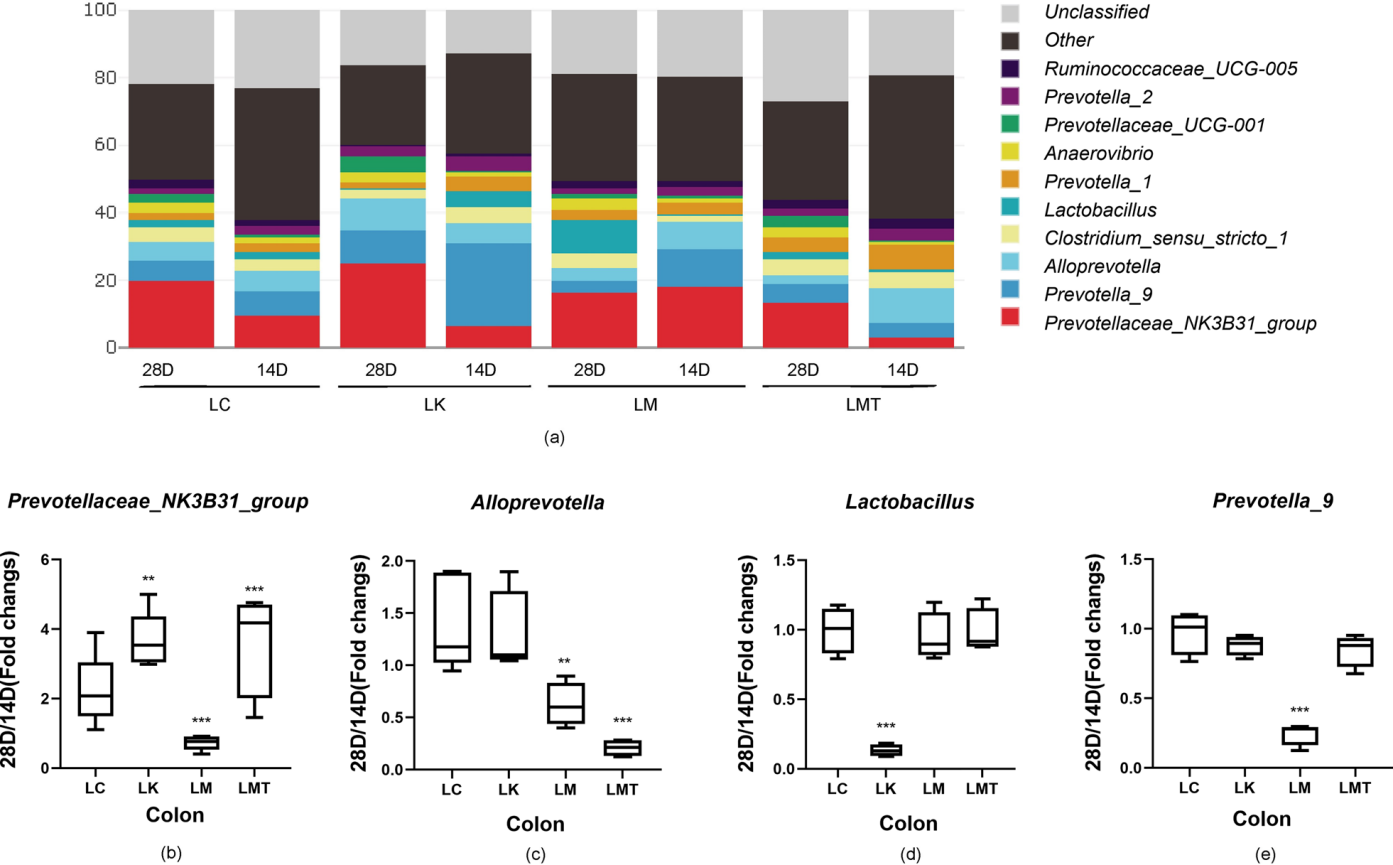
The composition of colon microbiota at the genus level. **a** The effect of the TCM formula on the composition of the colon microbiota at the genus level in the weaned piglets. **b** The fold change in the relative abundance of the genus *Prevotellaceae NK3B31* (28 d/14 d ratio). **c** The fold change in the abundance of the genus *Alloprevotella* (28 d/14 d ratio). **d** The fold change in the abundance of the genus *Lactobacillus* (28 d/14 d ratio). **e** The fold change in the abundance of the genus *Prevotellaceae 9* (28 d/14 d ratio) (*p < 0.05; **p < 0.02; ***p < *0.01*)

To reveal the effects of the TCM formula on the colon microbiota of piglets, 16S rRNA sequencing was carried out on the Illumina technology platform. From day 14 to day 28, the fold increases in the relative abundance of *Prevotellaceae NK3B31*(0.727, *p* < *0.001)*, *Alloprevotella* (0.946, *p* < *0.01*), and *Prevotella-9* (0.246, *p* < *0.001*) in the LM group were significantly lower than those in the other groups (Fig. 6), especially the fold increase of *Prevotella_9* abundance was the lowest. In addition, compared with the LK group (0.133), groups LM (0.946, *p* < *0.001*), LMT (0.984, *p* < *0.001*), and LC (0.997, *p* < *0.001*) showed greater fold increases in the relative abundance of *Lactobacillus* at 28 and 14 d, and *Lactobacillus* growth in the LK group was the slowest.

### Comparison of the predominant bacterial community structure in the caecum among the groups

**Fig. 7:**
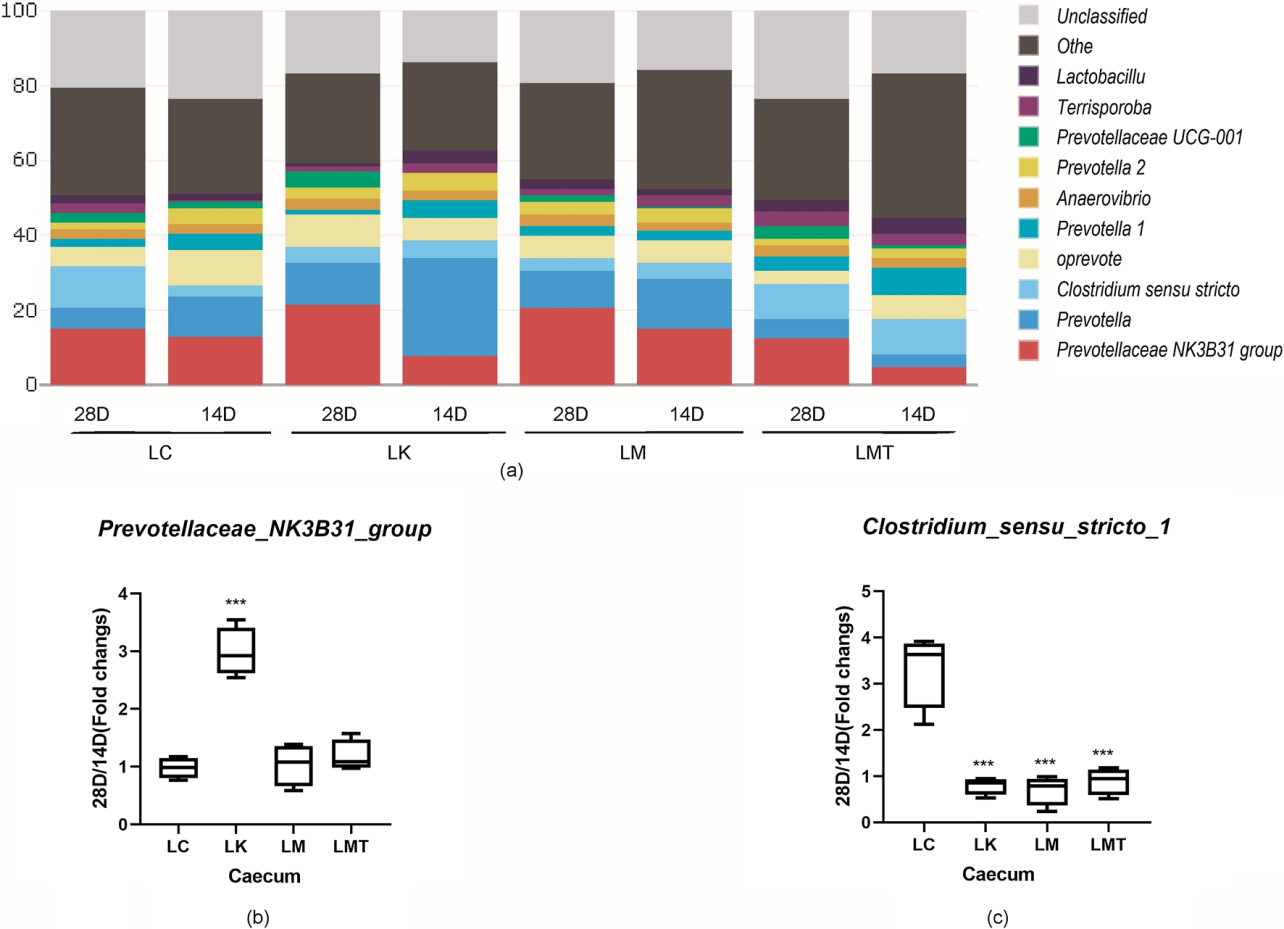
Composition of the cecum microbiota at the genus level. **a1** The effect of the TCM formula on the composition of the cecum microbiota at the genus level in the weaned piglets. **b** The fold change in the relative abundance of the genus *Prevotellaceae NK3B31* (28 d/14 d ratio). **c** The fold change in the abundance of the genus *Clostridium *sensu stricto* 1* (28 d/14 d ratio) (*p < 0.05; **p < 0.02; ***p < *0.01,*)

To uncover the effects of the *Coix seed* and *Lotus seed* on the bacterial community of the cecum in piglets, we collected cecum content for analysis on the 14th and 28th d of the experiment, and compared these data (Fig. 7). Groups LM and LMT featured lower abundance of *Clostridium* and *Prevotellaceae NK3B31* (LM:1.034, *p* < *0.001;* LMT:1.181, *p* < *0.001*)*.* The LK group showed a significant increase in *Prevotellaceae NK3B31* abundance (2.982, *p* < *0.001*) in the cecum of piglets. *Clostridium* in the LC group (3.327, *p* < *0.001*) was up to fourfold more abundant at 28 d than at 14 d, but groups LK (0.795), LM (0.698), and LMT (0.895) did not show obvious up-regulation.

### Effects of the formula based on the *Coix seed* and *Lotus seed* on the bacteria in the stomach

**Fig. 8:**
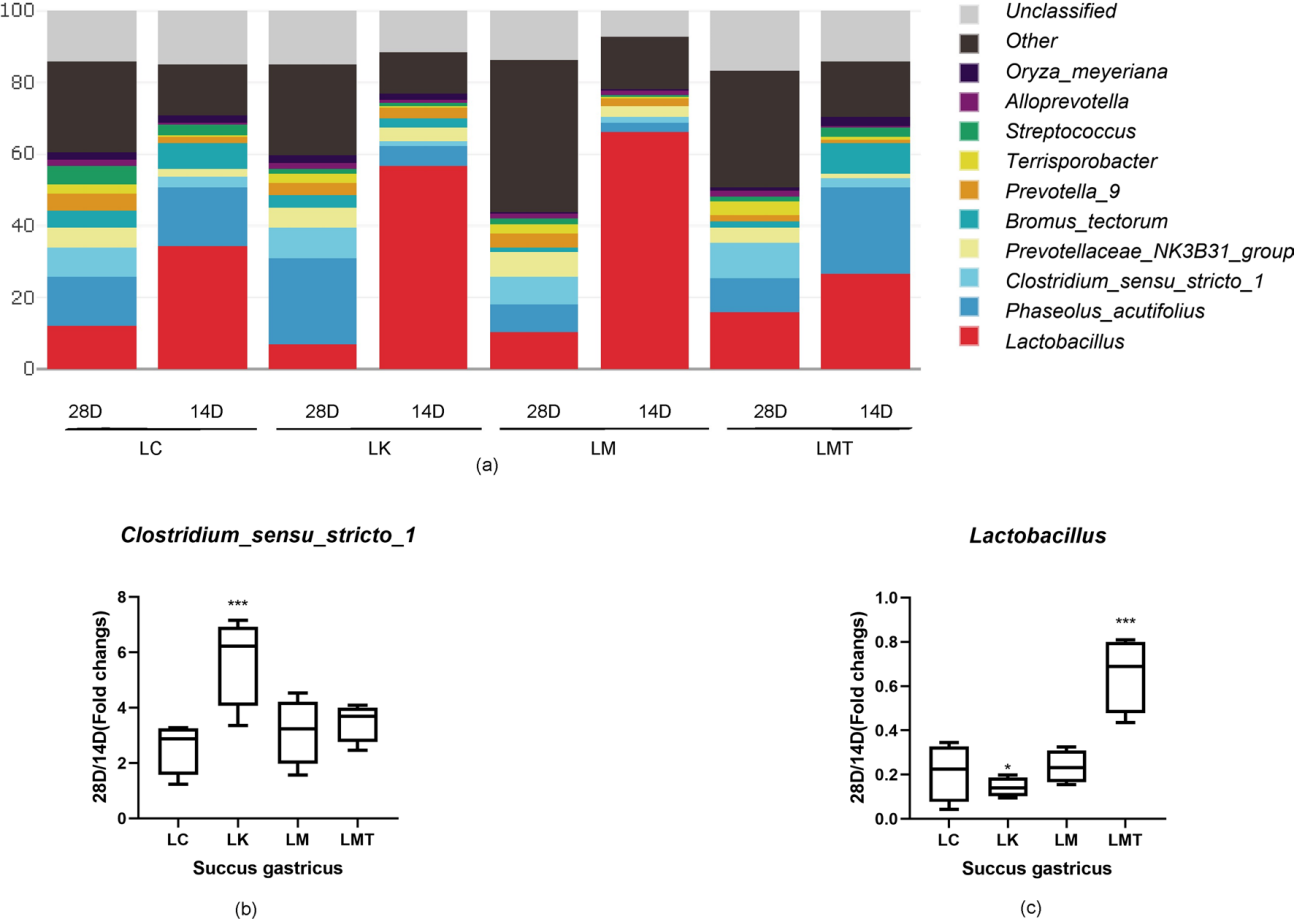
The composition of the succus gastricus microbiota at the genus level. **a** The effect of the TCM formula on the composition of the succus gastricus microbiota at the genus level in the weaned piglets. **b** The fold change in the relative abundance of the genus *Clostridium *sensu stricto* 1* (28 d/14 d ratio). **c** The fold change in the abundance of the genus *Lactobacillus* (28 d/14 d ratio) (*p < 0.05; **p < 0.02; ***p < *0.01*)

**Fig. 9:**
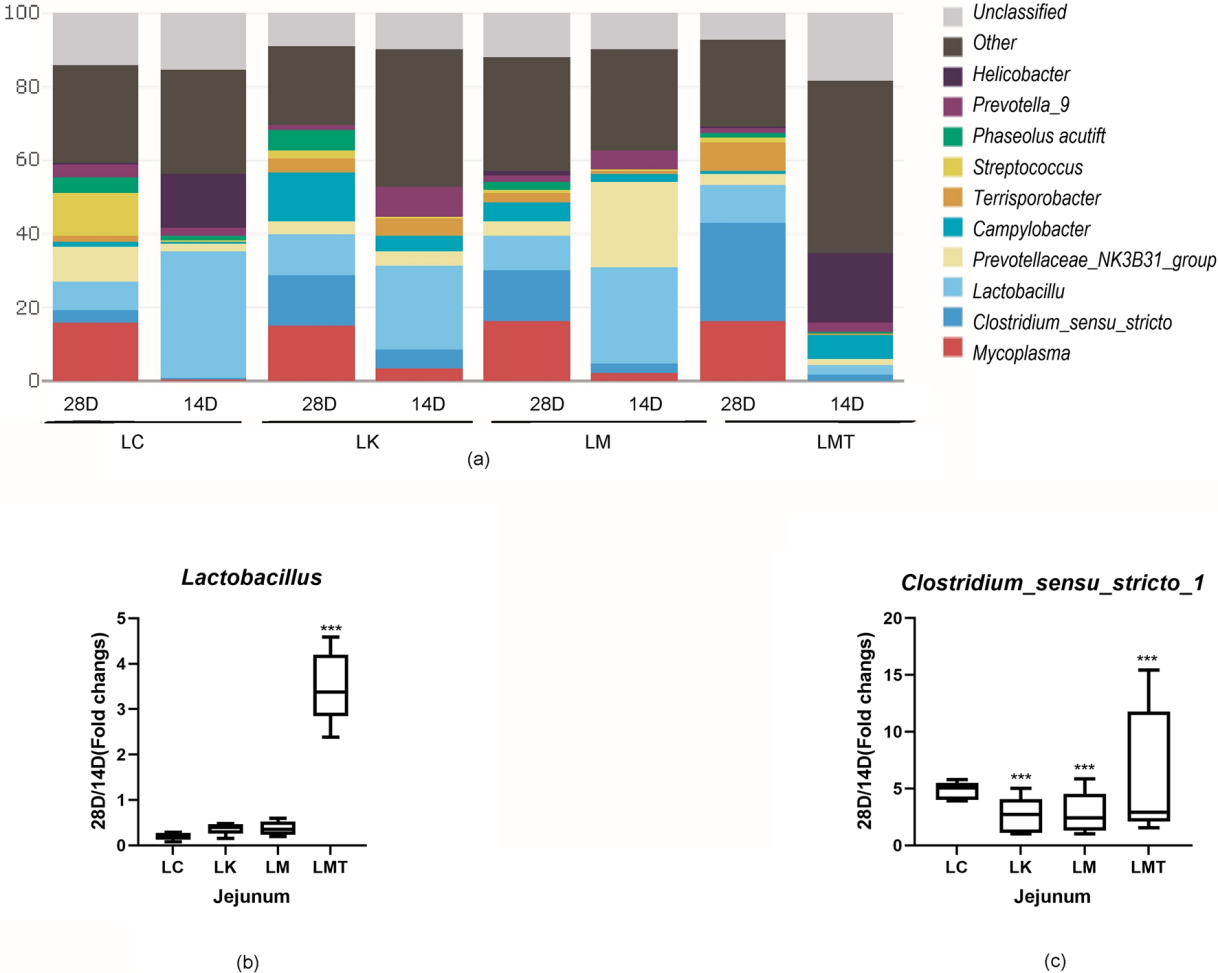
Composition of the jejunum microbiota at genus and phylum levels. **a** The effect of the TCM formula on the composition of the jejunum microbiota at the genus level in the weaned piglets. **b** The fold change in the relative abundance of the genus *Lactobacillus* (28 d/14 d ratio). **c** The fold change in the abundance of the genus *Clostridium *sensu stricto* 1* (28 d/14 d ratio) (*P < 0.05; **P < 0.02; ***P < *0.01*)

Microorganisms in the stomach and jejunum secrete various enzymes to promote the absorption of nutrients, and thus, the difference in the microbial community between the stomach and jejunum plays an important role in the health of piglets. Microbiota analysis of piglets’ stomach indicated that in the LMT group (0.655, *p* < *0.001*), the abundance of *Lactobacillus* enormously increased than other groups, thus the LK group (0.1430, *p* < *0.05*) was the lowest. The fold increase in *Clostridium_sensus_stricto_1* relative abundance was the highest in the LK group (5.742, *p* < *0.001*) (Fig. 8), and other groups not clearly differences each other. In this study, the abundance of *Lactobacillus* in the LMET group (3.493, *p* < *0.001*) enormously increased in the jejunum. Groups LC, LK, and LM clearly showed the down-regulation of *Lactobacillus* in the jejunum. And then, the fold increase of *Clostridium _sensus_stricto_1* relative abundance was the highest in the LC group with 4.810 (Fig. 9), and the abundance in LK(2.600, *p* < *0.001*) and LM (2.811, *p* < *0.001*) groups significantly decreased between days 14 and 28 (Fig. 9), but the LMT group has one sample which has higher record with 15.442, if the data is excluded, its average was 3.042 be closed the LK and LC groups.

## Discussion

TCM formulations as dietary supplements have positive effects on the growth performance of piglets: previous reports have demonstrated that dietary supplementation with the TCM herbs can increase average daily feed intake and ADG, improve the gain/feed ratio, and reduce the incidence of diarrhea in weaned piglets (Chen et al., 2007; Lin et al., 2013; Xu et al., 2012; Zhao et al., 2015; Jian et al., 2007). Here, our results suggest that the LMT group manifested a significant increase in ADG, and the FCR tended to diminish in these piglets as compared to that in the LC group, in agreement with previous research (Lai et al., 2015; Gui et al., 2007). This result can be explained as follows: the TCM formula extract is rich in starch, polysaccharides, and protein, which promoted the growth of piglets. Another important factor is that the TCM formula extract reverses the dysfunction of the spleen and stomach and disorders of water transport and corrects malnutrition. Nevertheless, in this study, we found that the FCR values of the LK group also followed a clearly decreasing trend approaching that of the LMT group. Therefore, appropriate addition of antibiotics can correct an intestinal imbalance and thus promote the growth performance of piglets (Nemechek et al., 2013).

pH levels in the gastrointestinal tract of piglets play an important role in the maintenance of normal physiological function of the digestive system and regulate the acid–base balance and electrolyte balance in the inner circumferential environment (Schnabel et al., 1982; Clark et al., 2010; Straw et al., 1991). Low pH in the stomach can accelerate the conversion of pepsinogen to pepsin and expansion and denaturation of feed protein; these processes are beneficial for the digestion of feed protein (Burnell et al., 1988). In addition, low pH in the stomach can slow down the gastric emptying rate, delay the residence time of feed in the stomach, and promote the digestion and absorption of nutrients (Clark et al., 1993). The present study also revealed that the pH values of the stomach were lower in groups LMT and LM than in groups LK and LC; these data are consistent with the growth performance mentioned above (Suiryanrayna et al., 2015).

It is known that an enormous number of exogenous and internal factors mediate the regulation of pH in the gastrointestinal tract. Among them, the diet is an important exogenous factor. According to the latest reports, stomach acidity is mainly affected by the buffering capacity of solid food and water (Meler A et al., 2012; Kong et al., 2009). In recent years, more and more studies suggest that herbs and extracts help to regulate pH of the gastrointestinal tract (Xu et al., 2009). In the present study, groups LMT and LM showed lower pH in the stomach of piglets, in line with previous research (Xu et al., 2009). The TCM formula and TCM formula extract acted as nutritional supplements that regulate gastrointestinal pH. Nevertheless, pH of the ileum in groups LM and LMT ended up at 6.8, which was clearly higher than pH in the LK and LC groups.

The gastrointestinal villi of piglets not only act as a tissue for food digestion and absorption but also play an important part in the fight against pathogenic bacteria and toxic substances present in the intestinal lumen (Yang et al., 2013). Therefore, ensuring the normal development and growth of intestinal villi is the key to improving the growth performance of piglets. Previous research suggests that weaning is substantial stress for piglets and is accompanied by a reduction in villus height and worsening of villus morphology, and the small intestine continues to deteriorate until the fifth day after weaning (Miller et al., 1986). To reduce the effects of stress on piglets, many authors have suggested that TCM supplements can increase villus height (reduce villous atrophy) and crypt depth (reduce crypt hyperplasia), and a lot of studies have indicated that some TCM formulations can promote the development and growth of intestinal villi (Yang et al., 2013). In this study, groups LM and LMT showed a subsequent increase in the villus height in the stomach and cecum the whole experiment; this finding is also consistent with the growth performance mentioned above (Shi et al., 2015).

According to the influence of the intestinal microflora on host health, it can be subdivided into probiotics, harmful bacteria, and opportunistic bacteria (Clemente et al., 2012). While probiotics such as *Bacteroidetes* and *Lactobacillus* dominate in the host intestine, the host is in a relatively healthy state. A large number of studies have shown that TCM can significantly alleviate intestinal microflora disorders, promote the growth of beneficial bacteria, inhibit the excessive propagation of harmful bacteria, and balance the abundance of these probiotics and pathogenic bacteria, thus maintaining a healthy environment in the intestinal tract (Li et al., 2010; He et al., 2007). For example, polysaccharides, an active ingredient of TCM formulations, can improve the intestinal environment, contribute to the growth of probiotics, and may play the role of a prebiotic, which is beneficial to the growth of probiotics (Wang et al., 2015). These prebiotics promote the growth of probiotics by changing the growth environment of the intestinal flora or by serving as a substrate, and some organic acids in TCM formulations act as pH buffers to maintain the stability of pH in the intestinal tract to provide a suitable living environment for probiotic proliferation (Modesto et al., 2009). In particular, herbal supplements such as ginseng, *Ganoderma lucidum*, *Lotus seed*, *Coix seed*, and *Codonopsis pilosula* can promote the growth of probiotics to a certain extent (Koo et al., 1994; Zeng et al., 2018; Liu et al., 2019). Their ingredients can accelerate the growth of probiotics (e.g., *Bifidobacterium*, *Lactobacillus*, and *Streptococcus thermophilus*) after entering the intestine (Li et al., 2010). In the present study, the TCM formulae used in groups LM and LMT are based on the *Lotus seed* and *Coix seed*, which are rich in prebiotics such as amino acids, polysaccharides, and starch(Xi et al., 2019; Xu et al., 2018). These formulations also significantly promote the proliferation of *Lactobacillus* in the colon, jejunum, and stomach.

In addition, TCM herbs can inhibit the growth of pathogenic microorganisms, especially the harmful bacteria in the intestinal tract (Wang et al., 2010; Xuan et al., 2014; Chandrakar et al., 2015). This study also proved that treatments LM and LMT significantly inhibited the growth of Prevotella, Alloprevotella, and Clostridium. On the contrary, in the LK group and LC group, there was an obvious increase in the relative abundance of microorganisms. Particularly, the LK group showed higher relative abundance of stomach *Clostridium. Clostridium spp.* belongs to firmicutes, which is a large class of normal facultative *anaerobic bacteria* in intestine. *Clostridium* mainly was consists of two types: harmful and beneficial. Dozens of *Clostridium* strains have been reported in previous studies. Some of them were used to prevent, diagnose and treat relative human diseases, and others were demonstrated to be related to diseases including antibiotic-associated diarrhea, pseudomenbraneouscolitis and so on. In this study, whether the genus *Clostridium* in LK group is a pathogenic microbe need to be demonstrated in the further study.

The TCM formula based on the *Coix seed* and *Lotus seed* not only reduces gastrointestinal pH and increases the villus height but also promotes the growth of probiotics. Additionally, it inhibits the proliferation of pathogenic microorganisms and thus improves production performance of piglets. The proposed nutritional supplement seems to be an appropriate substitute for antibiotics in piglets feed.

## Data Availability

The datasets generated during and/or analyzed during the current study are available from the corresponding author on reasonable request.
